# Sequence-based prioritization of i-Motif candidates in the human genome

**DOI:** 10.3389/fbinf.2025.1657841

**Published:** 2025-08-12

**Authors:** Veronica Remori, Michela Prest, Mauro Fasano

**Affiliations:** ^1^ Department of Science and High Technology, University of Insubria, Como, Italy; ^2^ Center of Neuroscience Research, University of Insubria, Busto Arsizio, Italy

**Keywords:** i-Motif, multiple sequence alignment, position-specific similarity matrix, prioritization, random forest

## Abstract

**Introduction:**

i-Motifs (iMs) are cytosine-rich, four-stranded DNA structures with emerging roles in gene regulation and genome stability. Despite their biological relevance, genome-wide prediction of iM-forming sequences remains limited by low specificity and high false-positive rates, leading to considerable experimental burden.

**Method:**

To address this, we developed a refined computational approach that prioritizes high-confidence iM candidates using a Position-Specific Similarity Matrix (PSSM) derived from multiple sequence alignments. The human reference genome (hg38) was scanned using a custom regular expression targeting cytosine-rich motifs, followed by scoring each sequence with the PSSM. Statistical significance was assessed via permutation testing, one-sided t-tests, Benjamini-Hochberg correction, and Z-scores.

**Results:**

This pipeline identified 37,075 candidate sequences (15–46 nucleotides) with strong iM-forming potential. Validation against experimentally confirmed iMs and known G-quadruplexes (G4s) demonstrated significant differences in alignment scores and sequence similarity, confirming structural specificity. A random forest classifier trained on nucleotide features further supported the distinctiveness of the candidates, achieving a high classification performance.

**Conclusion:**

This work presents a scalable and statistically robust method to enrich for biologically relevant iM sequences, providing a valuable resource for future experimental validation and the rational design of ligands targeting iMs to modulate gene expression in contexts such as cancer.

## 1 Introduction

DNA, the molecular blueprint of life, primarily exists in the canonical double helix of the B form (Travers and Muskhelishvili, 2015). However, beyond this classical structure, DNA is capable of adopting a variety of non-canonical conformations, including triplexes, cruciforms, G-quadruplexes (G4s), and i-motifs (iMs) (Abou Assi et al., 2018). Among these, iMs are four-stranded structures formed in cytosine-rich sequences, stabilized by hemi-protonated cytosine-cytosine (C:C+) base pairs (Luo et al., 2023). These structures are different from the more widely studied G4s, which are formed by guanine-rich sequences, and have recently gained attention due to their potential role in fundamental biological processes (Ban et al., 2024; Sen and Gilbert, 1988).

First identified in the early 1990s, iMs are known to form under mildly acidic conditions, a property that suggests their involvement in cellular environments with low pH, such as the nucleus or specific subcellular compartments (Kikuta et al., 2015). iMs are not merely theoretical; they have indeed been observed *in vivo*, where they are found in critical regions of the genome, including the promoter regions of oncogenes and in telomeric DNA, both of which are crucial for gene regulation and chromosomal stability (Peña Martinez et al., 2024). These structures can act as molecular switches, modulating gene expression by transitioning between stable and dynamic conformations. The ability of iMs to influence gene transcription makes them attractive targets for therapeutic intervention, particularly in cancer and other diseases linked to gene dysregulation (Deep et al., 2025).

Despite their promising biological roles, much remains unknown about iMs, particularly regarding their formation dynamics and recognition by proteins and small molecules. Identifying iM-forming sequences within the genome is a crucial step in advancing this research field. However, predicting which sequences are capable of adopting the iM conformation remains challenging, as current methods for genome-wide screening of iM candidates are still limited (Yu et al., 2024; Sengupta et al., 2024). A significant limitation of existing approaches is the generation of extensive candidate lists, which often lack sufficient specificity and can result in a high degree of computational and experimental burden. This gap in knowledge underlines the need for more refined methods to identify and prioritize potential iM-forming sequences. A focused list of high-confidence candidates would enable researchers to concentrate on the most biologically relevant sequences, facilitating a deeper understanding of their structural, functional, and therapeutic implications, and supporting the development of small molecules or ligands that can target these structures to modulate gene expression (Debnath et al., 2019).

To address this gap in existing methods, we propose an approach based on a Position-Specific Similarity Matrix (PSSM) derived from multiple sequence alignment to identify iM candidates.

## 2 Materials and methods

**FIGURE 1:**
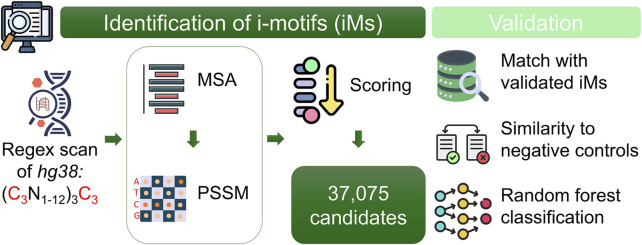
Overview of the computational pipeline for identifying potential i-motif (iM) sequences in the human genome. The genome was scanned on both strands using a custom regex to detect cytosine-rich motifs. Candidates were aligned per chromosome and strand using a multiple sequence alignment (MSA) method. A Position-Specific Similarity Matrix (PSSM) was constructed from the alignments to score and prioritize sequences. Validation was performed by comparing top candidates to experimentally confirmed iMs and G-quadruplexes, supported by statistical analyses and machine learning classification.

To identify and analyse potential DNA regions capable of forming iMs in the human genome, the employed method followed a systematic four-step approach. It integrated genome-wide scanning, sequence scoring, pattern refinement, and validation, ensuring comprehensive identification and analysis of iM-forming candidates (Figure 1). All the program codes used in this study were implemented in R (4.2.0), providing a flexible and efficient framework for data processing and analysis.

### 2.1 Identification of candidates

The initial step of the methodology involved the scanning of the *hg38* human genome reference (Li R. Y. et al., 2023). This reference genome was examined by both forward and reverse strands, ensuring the coverage of all genomic regions. The scanning process was performed using a custom-designed regular expression (regex) pattern to detect sequences that are likely to adopt iM structures. The regex pattern specifically searched for stretches of three consecutive cytosines (C), flanked by a variable sequence of 1–12 bases, denoted as “N” to represent any nucleotide (adenine, thymine, cytosine, or guanine). The used full pattern was (C_3_N_1-12_)_3_C_3_, which targeted genomic regions where four stretches of three cytosines were present. These sequences were highly indicative of potential iM formation due to their characteristic cytosine-rich structure (Abou Assi et al., 2018). This scanning process resulted in the identification of potential iM-candidates across the entire genome.

The next step involved evaluating the likelihood that these sequences would form stable iMs. This was accomplished by constructing a Position-Specific Similarity Matrix (PSSM) using multiple sequence alignment (MSA). To ensure the most accurate and reliable alignment, three different MSA algorithms were assessed: ClustalW (Larkin et al., 2007), ClustalOmega (Sievers et al., 2011), and DECIPHER (Wright, 2015). The optimal algorithm was selected through an empirical comparison based on three main criteria: the number of high-scoring iM candidates preserved after alignment, the minimization of gaps within the core motif region, and the consistency of alignment performance across both DNA strands. Once identified the optimal MSA algorithm, it was applied independently to each chromosome and strand to capture potential strand-specific variations in iM formation. Additionally, the three nucleotides flanking the candidate sequence on both sides were included in the alignment. The rationale behind applying MSA is that sequence conservation can act as a proxy for structural and functional relevance: motifs that are positionally conserved across multiple loci are more likely to reflect biologically stable iMs, as opposed to randomly occurring sequences. In this way, MSA helps reduce noise and highlight core features that may drive iM stability.

Nucleotide frequencies for each position in the alignment were then computed. For each position, the frequency of occurrence of each nucleotide (A, T, C, G) was calculated by determining the proportion of each present nucleotide relative to the total number of sequences in the alignment. These frequencies were then used to compute logarithmic scores, which represent the ratio between the observed frequency of each nucleotide at a given position and the expected frequency under a uniform distribution, where each nucleotide has an equal probability of appearing.

The PSSM was then built by incorporating the log-transformed scores for each nucleotide at every position in the alignment. This resulted in a quantitative score for each sequence, reflecting its occurrence frequency and, by extension, its potential to form stable iM structures.

More in detail, the score for each identified sequence was determined by summing the individual scores for each position, as derived from the PSSM:

$$S=\sum_{\left.i\right|S_{i}>0}S_{i}$$

where $S_{i}$ is the score for position i according to the PSSM. In cases where the score at a particular position was negative, it was disregarded to avoid penalizing longer sequence matches that may exhibit lower scores at specific positions. Moreover, no gap penalties were applied to prevent biasing shorter matches during the scoring process. Scores were not normalized by sequence length, as this would have artificially favored shorter motifs. Instead, by summing only positive position-specific scores, we avoided penalizing longer sequences while maintaining a fair comparison across motifs of varying lengths.

To assess the significance of the observed scores, we performed a 1,000-times random permutation of the sequence order, recalculating the score for each permuted sequence. This procedure allowed for the generation of a distribution of scores under the null hypothesis that the sequences are randomly distributed. Subsequently, a one-tailed t-test was performed to compare the observed scores with the distribution of randomly generated scores, which produced p-values for each sequence. The t-statistic for this test was computed by comparing the difference between the observed score and the mean of the randomly generated scores, adjusted for the standard deviation and the number of permutations.

In addition, for the analysis of multiple comparisons, the Benjamini-Hochberg (BH) procedure was accurately applied to adjust the p-values, thereby controlling the false discovery rate. Additionally, to quantify the deviation of the observed scores from the expected random distribution, the Z-score for each sequence was calculated. This Z-score provided a standardized measure of how much the observed score deviates from the mean of the random permutations, offering insight into the robustness of each sequence potential to form stable iM structures.

Finally, the sequences with higher scores were identified as having a larger probability of forming stable iMs, while those with lower scores were considered less likely to adopt this conformation. Therefore, the top 5% of the results, ranked by Z-score, were retained and considered as suitable candidates. Then, these sequences were annotated using the *GenomicRanges* library to add genomic information, such as the gene symbol and the gene type. For each candidate sequence, direct overlaps with gene bodies were first identified based on the Gencode v38 gene annotation. Sequences without direct overlap were annotated with their nearest gene, including the genomic distance to that gene, to ensure comprehensive genomic assignment. Each sequence was thus labeled as either “correct” (within gene body) or “nearest” (closest gene) to reflect annotation confidence. To further investigate the biological context of the predicted iM-forming sequences, genes were classified into three categories: immune-related, housekeeping, and other. Housekeeping genes were obtained from the curated list in the Molecular Signatures Database (MSigDB) (https://www.gsea-msigdb.org/gsea/msigdb/cards/HOUNKPE_HOUSEKEEPING_GENES) (Hounkpe et al., 2021). Immune-related genes were collected by integrating multiple sources, including InnateDB (https://www.innatedb.com/annotatedGenes.do?type=innatedb) (Breuer et al., 2013), and MSigDB “IMMUNE_SYSTEM_PROCESS” and INNATE_IMMUNE_SYSTEM signatures (https://www.gsea-msigdb.org/gsea/msigdb/cards/HOUNKPE_HOUSEKEEPING_GENES and https://www.gsea-msigdb.org/gsea/msigdb/human/geneset/REACTOME_INNATE_IMMUNE_SYSTEM.html). All gene symbols were unified and deduplicated before downstream analyses. Gene class annotations were then assigned to each candidate based on overlap with these curated gene sets. To assess whether the predicted iM-forming sequences were significantly enriched in immune-related or housekeeping genes, a contingency table was constructed summarizing the presence and absence of each gene category within the candidate set and the background genome. A Fisher’s exact test was then performed to evaluate the statistical significance of gene category enrichment.

### 2.2 Validation of candidates

To validate the list of iM-candidates, a subset of 285 sequences with high Z-scores was randomly selected. The iM formation of these sequences were confirmed by previous works (GSE227616), with data obtained from custom-designed microarrays aimed at studying DNA sequences capable of forming iM structures (Yazdani et al., 2023). In addition, 285 experimentally validated G4s detected on the *hg38* reference genome were considered as negative controls (Neupane et al., 2023).

To compare the two groups, the alignment score of G4s was computed according to the PSSM, considering all the possible alignments by shifting all the bases across every possible position. Thus, a Wilcoxon rank sum test with continuity correction was performed to investigate the difference between the two groups.

Subsequently, to compare the similarity between iM-candidates and G4s, both the Levenshtein and Jaccard distances were calculated (Berger et al., 2021; Baharav et al., 2020). Here, the set of positive controls was expanded by including all sequences in the list of candidates confirmed by the GSE227616 dataset. The Levenshtein distance measured the minimum number of single-character edits required to transform one sequence into another. The Jaccard similarity was computed by comparing shared k-mers (with k = 3) between sequences. These measures allowed the evaluation of the similarity between the iM-candidates and both the positive (iM) and negative (G4) sequences.

In addition, with the aim to strengthen the validation, a random forest model was trained using features derived from the nucleotide composition of the sequences to classify the sequences into positive or negative (Chen and Ishwaran, 2012). The dataset consisted of both experimentally validated iM-forming sequences (positive samples) and G4 sequences (negative samples), along with additional randomly generated negative sequences.

The randomly generated negative sequences were designed to avoid forming iMs by excluding sequences containing the “CCC” triplet. A total of 1,000 such sequences were generated with lengths ranging from 15 to 50 nucleotides, ensuring that they could not form iMs. These sequences were then combined with the existing dataset of validated iM and G4 sequences, resulting in a balanced dataset for training.

For each sequence, the nucleotide frequencies (C, G, A, T), and the sequence length were considered as features. The nucleotide frequencies were obtained by counting the occurrences of each nucleotide within the sequence, while the sequence length was simply the total number of nucleotides in each sequence. These features were used as inputs for the random forest model. The dataset was split into training (70%) and testing (30%) subsets. The model was evaluated using the test set, and performance metrics, including accuracy, precision, recall, and the area under the curve (AUC) for a receiver operating characteristic (ROC) analysis were calculated.

To optimize the model performance, a hyperparameter tuning was performed using 10-fold cross-validation and a grid search over the number of variables considered at each split (*mtry*). The best hyperparameters were selected, and the model was retrained using the optimal settings.

A confusion matrix was used to evaluate the final model performance, and the ROC curve was plotted to assess the model capability to discriminate between positive and negative classes. The final model was used to make predictions on a separate test dataset, which consisted of previously unseen sequences inside the final list of candidates.

## 3 Results

### 3.1 Identification of candidates

**FIGURE 2:**
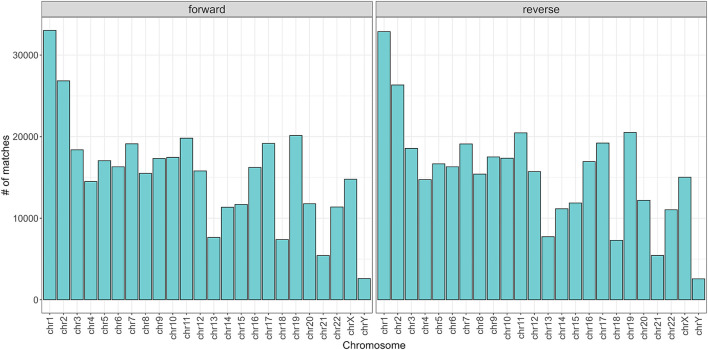
Number of matches divided by chromosome and strand.

**FIGURE 3:**
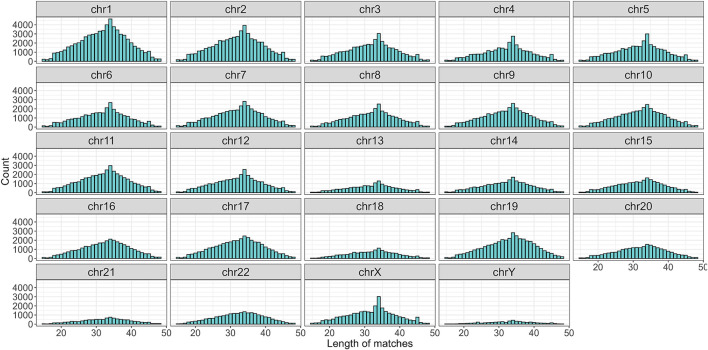
Distribution of match length divided by chromosomes.

To begin with, the regex matching across the *hg38* reference genome retrieved 742,510 sequences, whereof 370,558 on the forward strand, and 371,952 on the reverse strand. Figure 2 shows the number of matches for each chromosome, separated by strand. Chr1 and Chr2 exhibited the highest number of matches, while Chr13, Chr18, Chr21, and ChrY displayed a comparatively lower frequency of matches. Furthermore, the number of matches was almost equally distributed between the forward and reverse strands. The lengths of the matches ranged from 15 to 48 bases, with a mean of 32, a median of 33, and a standard deviation of 7. The distribution of the match lengths was similar across all the chromosomes (Figure 3).

Secondly, the best MSA algorithm was evaluated to be used with DNA sequences of different lengths. ClustalW and DECIPHER produced more symmetrical alignments with fewer gaps in the alignment of a subset of 10,857 matches (Chr 21) without distinguishing between strand orientation or sequence length, while ClustalOmega introduced more gaps and had alignments extending up to 200 positions (Supplementary Figure S1). When forward and reverse strands were separated, the DECIPHER performance became less symmetrical, indicating a larger sensitivity to strand orientation compared to ClustalW, which maintained a more consistent performance across both strands (Supplementary Figure S2). Additionally, ClustalW introduced fewer gaps than both ClustalOmega and DECIPHER, independently on the sequence length or quantity (Supplementary Figure S3). Consequently, ClustalW was rigorously employed to align all the sequences divided by strand and chromosome. In addition, to check for conserved motives before or after the iM, the alignment took into account also the three nucleotides before and after each match.

**FIGURE 4:**
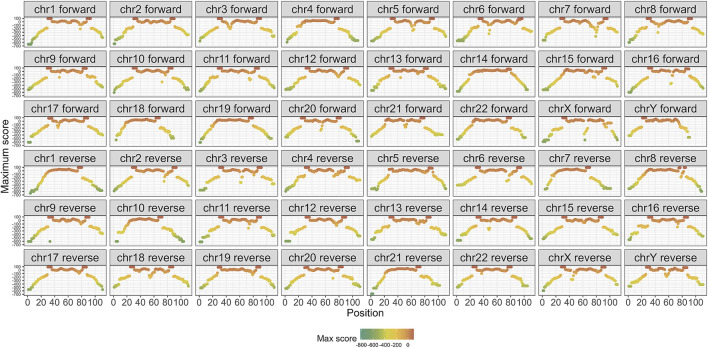
Maximum score of the PSSM based on strand and chromosome. Red: 100% of consensus.

Subsequently, the PSSM was computed for each alignment. Figure 4 presents the maximum PSSM score for each position within the alignment, categorized by strand and chromosome. The scores were rescaled as percentages, with 100% representing a fully conserved region. Notably, the central positions of the alignment exhibited the highest consensus, whereas the flanking positions showed a lower conservation. Conserved regions were marked by consecutive red dots. This per-chromosome and strand-specific breakdown reflects the fact that the multiple sequence alignments (MSAs) and corresponding PSSMs were constructed independently for each chromosome and strand. Reporting the scores at this resolution allows us to assess alignment quality and conservation patterns within the exact context in which scoring was performed, ensuring methodological transparency and avoiding biases introduced by global aggregation.

**FIGURE 5:**
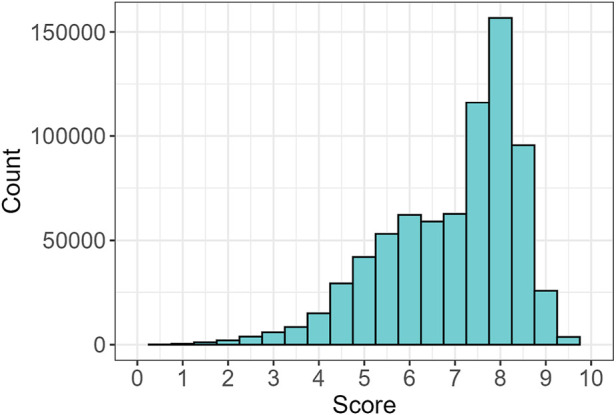
Distribution of scores for all the identified sequences.

Next, each sequence was scored to quantify the extent of conservation. The majority of sequences exhibited high scores (Figure 5). To evaluate the statistical significance of the observed scores, we performed a random permutation of the sequence order 1,000 times, recalculating the scores for each shuffled sequence. After verifying the normality of the random distributions, a one-sided t-test was performed to compare the observed scores with the distribution of randomly generated scores, producing p-values for each sequence. Next, the BH correction was applied, yielding that 99.9% of the sequences had scores significantly higher than the random distribution, suggesting that the great majority were different from random expectations (Supplementary Figure S4a). Additionally, the Z-score was calculated for each sequence to further assess the significance of the observed scores. The distribution of Z-scores is shown in Supplementary Figure S4b, providing an overview of the relative deviation of each sequence from the mean of the random distributions. Sequences with higher Z-scores indicate stronger evidence of being distinct from the random distribution.

**FIGURE 6:**
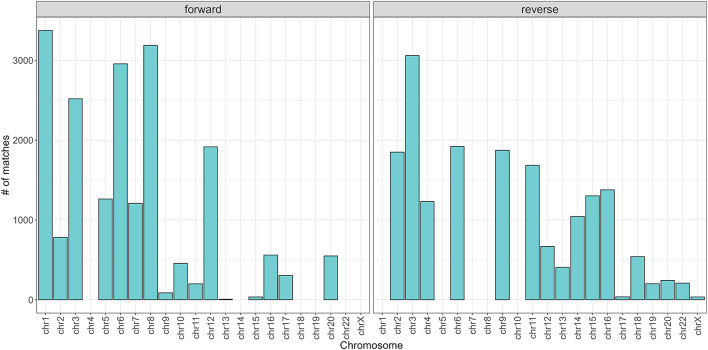
Number of sequences divided by chromosome and strand after the cutoff. Note that chr21 and chrY are no longer present.

**FIGURE 7:**
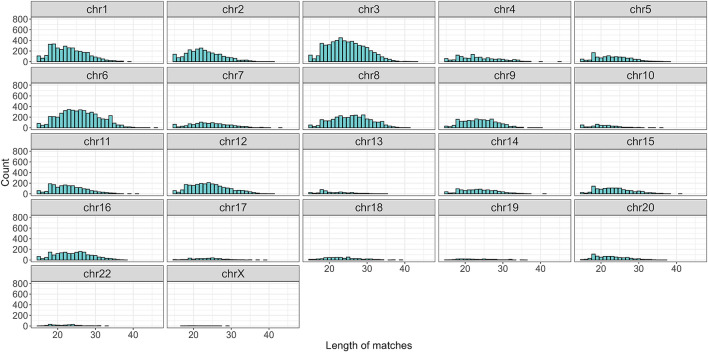
Distribution of sequence length divided by chromosomes after the cutoff. Note that chr21 and chrY are no longer present.

Finally, a threshold was applied by selecting only those sequences with an adjusted p-value below 0.05. Moreover, to control the False Discovery Rate (FDR) more rigorously, the selection was further refined by retaining only the top 5% of the results, ranked by Z-score. After applying the cutoff, a total of 37,075 sequences were selected, with 19,396 originating from the forward strand and 17,679 from the reverse strand (Figure 6; Supplementary Table S1). The distribution of matches differed between the two strands, with lengths ranging from 15 to 46 nucleotides and an average of 24. Noticeably, the match length distribution varied across chromosomes (Figure 7).

**FIGURE 8:**
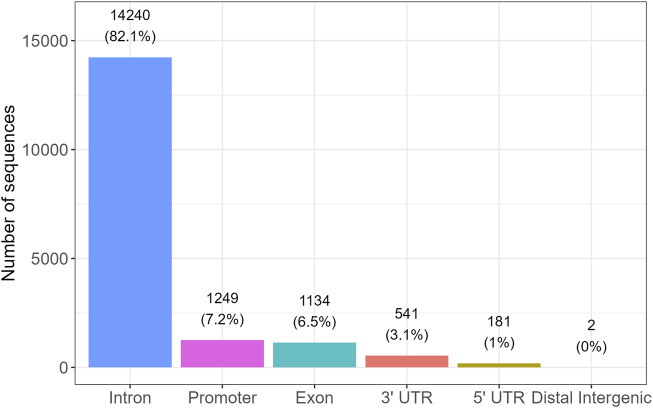
Distribution of confidently annotated i-motif candidate sequences across genic regions. Bar plot showing the number of high-confidence iM-forming sequences located in specific genomic regions, based on gene annotation. The majority of sequences were found within introns (82.1%), followed by promoter regions (7.2%), exons (6.5%), 3′untranslated regions (3.1%), and 5′UTRs (1.0%). Only two sequences were located in distal intergenic regions. These findings suggest a strong enrichment of iM-forming sequences in intragenic and regulatory regions.

Among these, 17,347 sequences were annotated as “correct,” indicating direct overlap with gene bodies, while the remaining sequences were annotated as “nearest,” corresponding to assignment to the closest gene based on genomic distance. Among the sequences annotated as “correct,” the genomic distribution was as follows: 14,240 (82.1%) were located within introns, 1,249 (7.2%) in promoter regions, 1,134 (6.5%) in exons, 541 (3.1%) in 3′UTRs, 181 (1.0%) in 5′UTRs, and 2 sequences (<0.1%) in distal intergenic regions (Figure 8). A Kruskal-Wallis test excluding distal intergenic sequences (n = 2) showed no significant differences in alignment scores among these genic regions (p = 0.36), indicating comparable score distributions across introns, promoters, exons, and UTRs.

To further explore the biological relevance of the predicted iM-forming sequences, enrichment in immune-related and housekeeping genes was evaluated. A contingency table comparing the occurrence of immune and housekeeping genes within the candidate set versus the background genome was constructed, and a Fisher’s exact test was performed. The test revealed a highly significant enrichment of immune-related genes among the predicted candidates (p < 2.2e-16), with an estimated odds ratio of 48.6 (95% confidence interval: 31.6–78.3), indicating that immune genes were substantially overrepresented in the iM candidate list compared to housekeeping genes.

### 3.2 Validation of candidates

To evaluate the iM-candidates, data from the GSE227616 dataset were compared with the identified candidates. Among the sequences present in the dataset and experimentally validated more than once, only 1,685 could be uniquely mapped and identified using a gene symbol, allowing a direct comparison with the list of iM-candidates. Notably, 1,286 of the identified candidates were also present in the dataset, further supporting their relevance.

A list of 285 sequences with high Z-scores was selected and was compared to a set of 285 experimentally validated G4s, which served as negative controls. The alignment scores for the second group were calculated using the PSSMs, considering all possible base shifts at each position. A Wilcoxon rank sum test with continuity correction was performed to assess the statistical difference between the two groups, yielding a W statistic of 81,225 and a p-value of 2.2 · 10^−16^ (Supplementary Figure S5). These results indicate that the two groups were significantly different.

To evaluate the sequence-level similarity, Levenshtein distances were computed between iM candidates and both positive (including 1,286 sequences validated in the GSE227616 dataset) and negative control sets (Supplementary Figure S6). All candidates were more similar to the positive set than to the negative one, resulting in their classification as iM-like. A Wilcoxon rank-sum test confirmed a highly significant difference in similarity distributions (W = 763,587, p < 2.2 · 10^−16^), indicating a strong shift toward the iM profile.

To further assess the iM-like nature of the candidate sequences, we computed k-mer-based Jaccard similarity scores (k = 3) between each candidate and both positive and negative control sets. Each candidate was assigned the maximum similarity score obtained against sequences in each control group. A ROC analysis was then performed to determine the optimal discrimination threshold, yielding an AUC of 1.0 and an optimal Jaccard similarity cutoff of 0.5. Using this threshold, sequences were classified as “positive” if they exhibited greater similarity to the positive set than to the negative controls. Notably, all candidate sequences exceeded the threshold and were classified as positive, reinforcing their strong resemblance to known iM-forming sequences. The distribution of similarity scores further supported this distinction, with a clear shift toward higher similarity with the positive group (Supplementary Figure S7).

**FIGURE 9:**
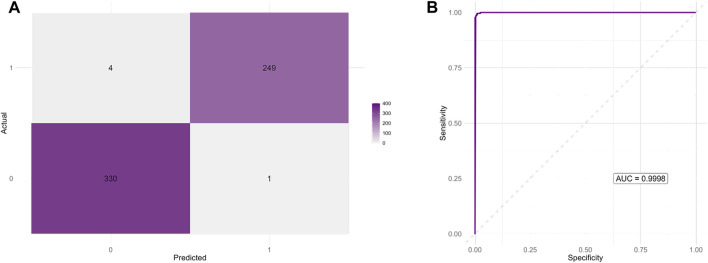
Performance of the Random Forest classifier distinguishing iM-forming from non-iM-forming sequences. **(A)** Confusion matrix showing the classifier predictions on the independent test set. The model achieved a high classification accuracy with minimal false positives and false negatives. **(B)** Receiver Operating Characteristic (ROC) curve for the same test set. The area under the curve (AUC = 0.9998) indicates near-perfect discriminative power between positive and negative classes.

An additional negative control group consisting of 1,000 randomly generated DNA sequences lacking cytosine triplets was included to further assess the iM-like characteristics of the candidate sequences. These sequences, together with validated iM-forming (positive) and G4-forming (negative) sequences, were used to train a Random Forest classifier based on nucleotide frequencies and sequence length. After 10-fold cross-validation and hyperparameter tuning (optimal *mtry* = 3), the final model achieved an accuracy of 99.32%, sensitivity of 99.10%, specificity of 99.60%, and balanced accuracy of 99.35% on an independent test set. The area under the ROC curve was 0.9998, indicating near-perfect discrimination between iM-forming and non-iM-forming sequences (Figure 9). The feature importance analysis identified cytosine frequency as the most informative variable, followed by guanine content and sequence length. When applied to the candidate sequences, the model classified 99.77% of them as positive, further supporting their strong similarity to validated iM-forming sequences and their clear separation from both biological and artificial negative controls (Supplementary Figure S8).

## 4 Discussion

In this study, a computational approach was developed to identify a list of potential iM-forming sequences across the human genome. The method leveraged a Position-Specific Similarity Matrix (PSSM) derived from multiple sequence alignment (MSA) to systematically detect genomic regions with a high propensity to adopt iM structures. The approach incorporated stringent statistical validation, including random permutation tests and Z-score calculations, ensuring that the observed patterns were not due to random chance. Furthermore, the analysis considered strand- and chromosome-specific variations, providing a comprehensive view of iM formation across different genomic contexts. The results revealed a significant enrichment of iM-forming sequences, reinforcing their potential biological relevance and suggesting a non-random genomic distribution.

The initial genome-wide search in the human reference genome (*hg38*) using a regex pattern matching identified a total of 742,510 candidate sequences, which were almost evenly distributed between the forward and reverse strands. This demonstrated the widespread presence of potential iM-forming regions across the genome, consistent with recent literature (Martella et al., 2022). To refine the dataset, a statistical filtering process was applied using adjusted p-values and Z-scores. Following the Benjamini-Hochberg (BH) correction for multiple comparisons, 99.9% of the sequences were found to be statistically significant, indicating a high probability of forming stable iM structures. These results validated the robustness of the method and confirmed a non-random distribution. As a result, a set of 37,075 high-confidence sequences (5% of candidate sequences) was selected according to their Z-score. These sequences ranged in length from 15 to 46 nucleotides, with a mean length of 24 nucleotides. The match length distribution varied across chromosomes, suggesting that iM formation may strongly depend on the chromosomal context. Accordingly, iM-forming sequences may act as boosters of genomic instability (Duardo et al., 2023). It is worth noting that chromosomes 21 and Y did not contain any candidates on either the forward or the reverse strand. The distribution also varied with the strand direction, consistent with iM- and G4-forming sequences being complementary (Tao et al., 2024).

Furthermore, a comparison between the identified iM-candidates and a set of experimentally validated G4 sequences revealed a significant difference in their alignment scores, as confirmed by the Wilcoxon rank sum test. This finding supports the hypothesis that iMs and G4s are structurally independent entities with distinct sequence characteristics and alignment profiles (Chu et al., 2019). Recent reports have shown that iM structures are frequently found near G4-forming regions, highly transcribed genes, and genes expressed during the G0/G1 phase, emphasizing their non-random distribution and role in genomic organization (Peña Martinez et al., 2024). The clear separation between the two groups suggests that the approach successfully used here identified iM-specific sequences, distinct from those that form G4 structures. To further validate the identified iM-candidates, they were compared to known iM-forming sequences listed in the Gene Expression Omnibus database (GSE227616). Among the sequences in the dataset, 1,286 of the identified iM-candidates were present (76.3% of the list of experimentally validated and uniquely identified iMs), providing additional evidence for the reliability of the method. As a non-exhaustive example, the iM in the *HRAS* oncogene was included in the list of candidates: it is known to form a double-hairpin structure and to play a crucial role in regulating *HRAS* gene expression, a key player in cell proliferation pathways and cancer progression (Li K. S. et al., 2023). In addition, the iM structures upstream of the apoptosis regulator *BCL2* gene were retrieved (Kendrick et al., 2014).

Moreover, the list of 1,286 verified iMs was further employed as a positive control to validate the remaining 35,789 candidates. Sequence-level similarity was evaluated using the Levenshtein distances between the 35,789 iM candidates and both positive and negative control (the 285 G4s) sets. All candidate sequences exhibited lower distances, and thus greater similarity, to the positive set compared to the negative controls. This consistent trend led to their preliminary classification as iM-like. Statistical validation using a Wilcoxon rank-sum test confirmed a highly significant difference in the distributions of similarity scores, highlighting a marked shift toward the iM sequence profile. In addition, a k-mer-based Jaccard similarity analysis (k = 3) was performed. Each candidate sequence was compared to both positive and negative control groups, and the highest similarity score within each group was retained. Again, all candidates were classified as positive, having higher similarity to the validated iM-forming sequences than to the negative controls. The complete overlap of candidate sequences with the iM profile, and their clear distinction from G4-forming sequences, underscores the structural specificity of the identified motifs. This observation supports the notion that i-motif structures represent a distinct class of non-canonical DNA elements, potentially associated with unique regulatory functions that are not redundant with those of G-quadruplexes. The mutual exclusivity observed in sequence similarity reinforces the idea that iMs and G4s are not functionally redundant but rather operate in complementary but distinct genomic contexts.

To further assess the biological relevance of the candidate iM sequences, we applied a machine learning approach trained on validated iM-forming sequences, G4s, and randomly-generated DNA controls lacking cytosine-rich motifs. The classifier consistently distinguished iM-like sequences from both biological and “synthetic” negatives, reinforcing the idea that the candidates are not only structurally consistent with iMs, but also occupy a unique compositional and functional space within the genome. The strong predictive power of cytosine content, which emerged as the dominant feature in the model, aligns with the well-established sequence dependency of i-motif formation (Zhou et al., 2013; Abou Assi et al., 2018; Guédin et al., 2010). These results further support the classification of iMs as a distinct class of regulatory elements with specialized, non-overlapping roles relative to G-quadruplexes (Abou Assi et al., 2018; Guédin et al., 2010).

While G4s may facilitate transcriptional pausing or recruitment of transcription factors (Hänsel-Hertsch et al., 2016; Siddiqui-Jain et al., 2002), iMs could mediate repression or dynamic structural transitions in DNA during replication or repair (Zhou et al., 2013; Abou Assi et al., 2018; Kang et al., 2014). Their non-overlapping sequence preferences and structural constraints (Abou Assi et al., 2018; Guédin et al., 2010; Kang et al., 2014) suggest a complementary, layered regulation of genomic processes, potentially with tissue-specific or disease-relevant implications (Roxo and Pasternak, 2025). Consistent with this, enrichment analysis revealed a significant overrepresentation of immune-related genes among the predicted iM-forming sequences compared to housekeeping genes, suggesting a potential link between iM formation and immune system regulation. In line with this hypothesis, we observed that a large majority of high confidence iM candidates mapped to non-coding regulatory regions within genes. Specifically, over 82% of the confidently annotated iMs were located in intronic regions, followed by promoter regions (7.2%), exons (6.5%), and untranslated regions (4.1%). Only a negligible fraction (<0.01%) were found in distal intergenic areas. This enrichment near or within gene loci reinforces the proposed role of iMs in transcriptional and co-transcriptional regulation. It also supports the notion that iMs do not occur randomly in the genome but rather tend to cluster in regions where dynamic DNA structures can influence gene activity. Differential formation of iMs has been reported in certain cancer types and neurodegenerative disorders (Roxo and Pasternak, 2025; Wu et al., 2025), supporting their potential as biomarkers or therapeutic targets in precision medicine (Brown and Kendrick, 2021). The resulting list of potential iM-forming regions now offers a valuable resource for future experimental validation. Despite the growing interest in iMs, very few computational tools are currently available for their genome-wide prediction. Among these, iM-Seeker represents an important contribution, offering a flexible graph-based framework for motif detection and a machine learning strategy trained on experimental data. Its design allows users to explore a wide landscape of potential iM-forming sequences and assign folding probabilities and stability scores. Our approach is complementary in scope: while iM-Seeker emphasizes broad detection and flexible modeling, our method focuses on sequence conservation and statistical rigor to prioritize a compact and high-confidence set of candidates. Notably, our final list overlaps entirely with iM-Seeker’s predictions but constitutes only about 9.67% of its total output, thus offering a more selective entry point for experimental follow-up. These candidates could be experimentally tested *in vitro* and *in vivo* to confirm their ability to form iM structures, particularly in the context of gene regulation, where such structures may play critical roles in modulating gene expression (Zanin et al., 2023).

These findings are consistent with the increasing literature highlighting the significance of iMs in key genomic regions, such as the promoter regions of oncogenes and telomeric DNA, where iMs may play pivotal roles in regulating chromosomal stability and gene expression. For instance, natural i-motif structures are predominantly found in the promoter regions of various oncogenes, suggesting their involvement in gene regulation and their potential as therapeutic targets in cancer therapy (Luo et al., 2023). Their transcriptional regulatory roles in these regions make them promising therapeutic targets for disrupting oncogenic signaling. Recent discoveries, including the identification of a specific i-motif antibody, are driving advancements in this field (Brown and Kendrick, 2021).

Overall, these findings underscore the utility and robustness of the proposed computational framework in generating a high-confidence set of iM-forming sequence candidates. By integrating motif-based detection with alignment-informed scoring and rigorous statistical validation, the method offers a scalable and biologically meaningful strategy for genome-wide iM prediction. This set of candidates serves as a valuable resource for guiding experimental studies aimed at investigating the structural and functional roles of i-motifs.

Future work will focus on validating these sequences in relevant cellular systems, with particular attention to their involvement in transcriptional regulation, replication dynamics, and genome stability. To this end, several complementary experimental approaches could be employed to validate iM formation and function. Biophysical methods such as circular dichroism (CD) spectroscopy, UV absorbance melting, and nuclear magnetic resonance (NMR) spectroscopy are widely used to confirm the formation of i-motif structures under physiological conditions, particularly at slightly acidic pH or under molecular crowding (Alves et al., 2025). High-throughput techniques such as iMab-based immunoprecipitation sequencing (iM-IP-seq) and DNA microarrays have recently enabled large-scale experimental profiling of iM-forming regions in human cells (Ruggiero et al., 2025). Integrating these datasets with our predictions could offer a powerful validation pipeline. Furthermore, overlaying our candidate loci with ChIP-seq profiles of transcription factors or replication origin data could shed light on the regulatory potential of i-motifs in specific genomic contexts (Ma et al., 2012). In the long term, this catalog of candidate iMs may also guide the rational design of small-molecule ligands to selectively stabilize or disrupt i-motif structures in gene promoters, a promising avenue for therapeutic modulation in diseases such as cancer.

## Data Availability

The original contributions presented in the study are included in the article/Supplementary Material, further inquiries can be directed to the corresponding author.
