# Supplementary material for: Sequence-based prioritization of i-Motif candidates in the human genome
Source: Front Bioinform. 2025 Aug 12;5:1657841. doi: 10.3389/fbinf.2025.1657841 (PMC12378704; doi:10.3389/fbinf.2025.1657841)
Supplement: Supplementary file 1 [file DataSheet1.PDF]

Supplementary Figures

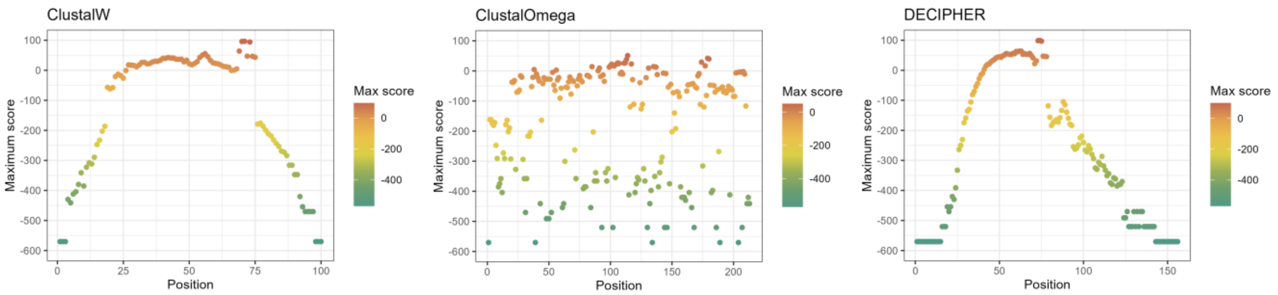

Supplementary Figure 1. Maximum score of the PSSM based on the alignment of a subset of 10,857 matches (Chr 21) without distinguishing between strand orientation or sequence length. ClustalW and DECIPHER produced more symmetrical alignments with fewer gaps, while ClustalOmega introduced more gaps and had alignments extending up to 200 positions.

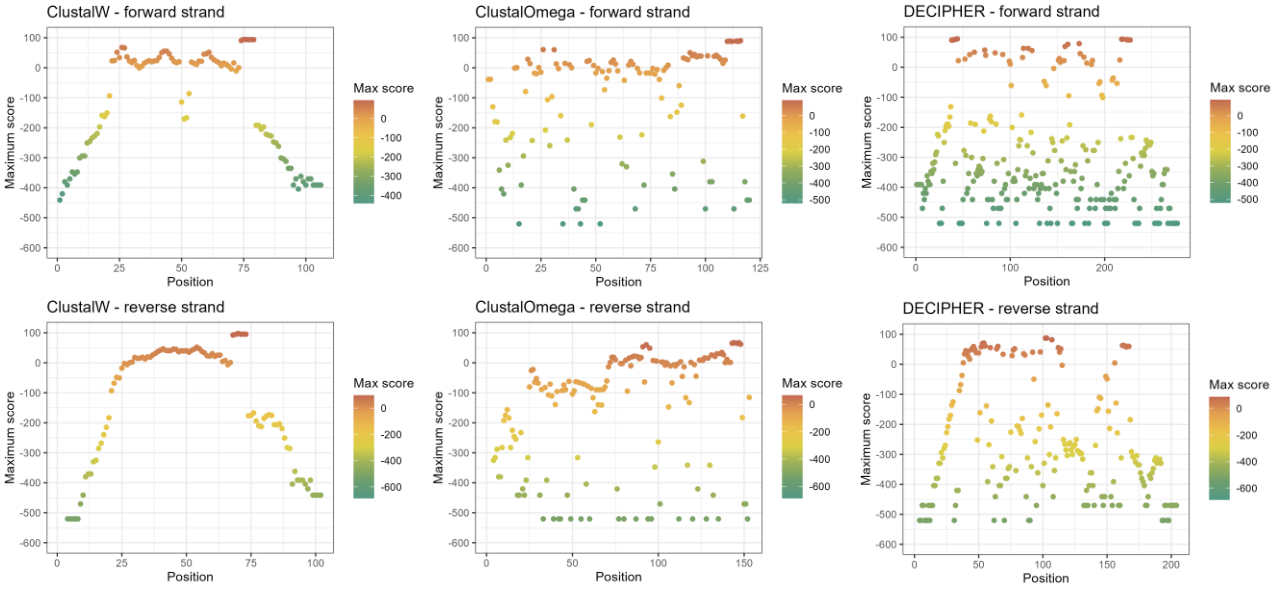

Supplementary Figure 2. Maximum score of the PSSM based on the alignment of a subset of 10,857 matches (Chr 21) separating by strand orientation. DECIPHER's performance became less symmetrical, indicating greater sensitivity to strand orientation compared to ClustalW, which maintained more consistent performance across both strands.

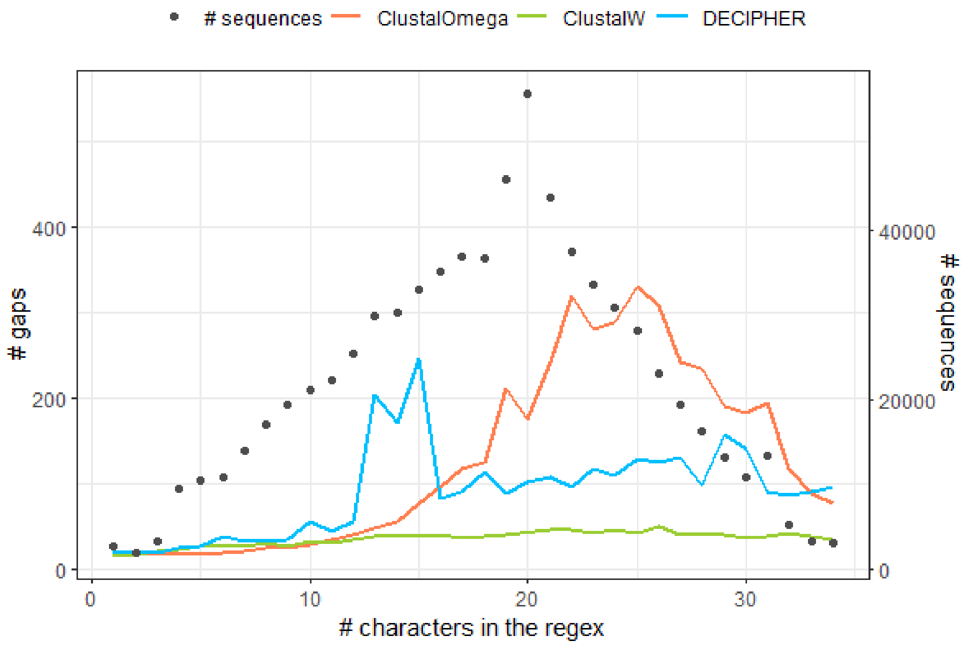

Supplementary Figure 3. Number of gaps introduced in the MSA of a subset of 10,857 matches (Chr 21) considering matches of the same length. ClustalW introduced fewer gaps than both ClustalOmega and DECIPHER, independent of sequence length or quantity.

a)

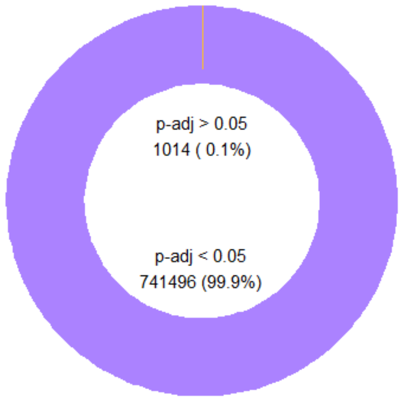

b)

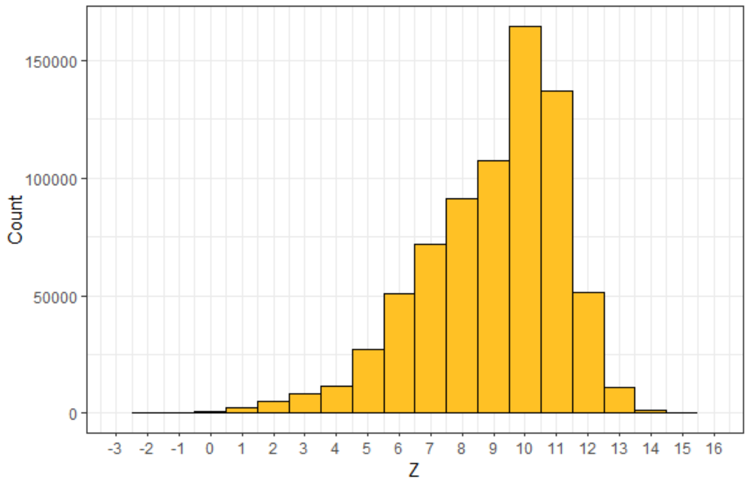

Supplementary Figure 4: a) Percentage of adjusted p-value after BH correction. **b)** Distribution of Z-scores.

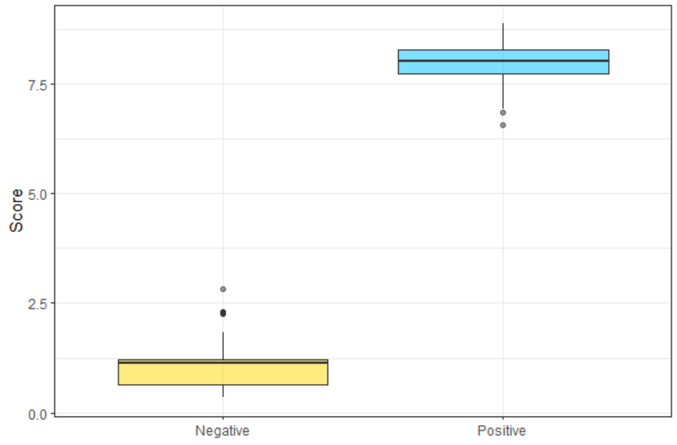

Supplementary Figure 5: Comparison of scores among two groups: positive = i-motif candidates, negative = G-quadruplexes.

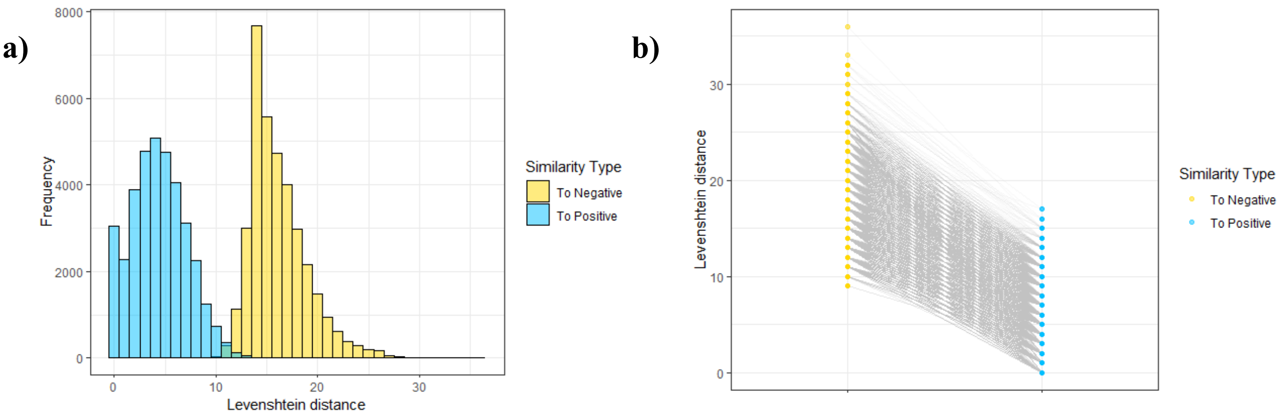

Supplementary Figure 6: a) Distribution of Levenshtein distances between candidate sequences and positive (light blue) or negative (yellow) controls. **b)** Paired Levenshtein distances for each candidate sequence relative to positive and negative control sets. Each line represents a single sequence, highlighting the consistent shift toward lower distance, thus greater similarity with the positive set.

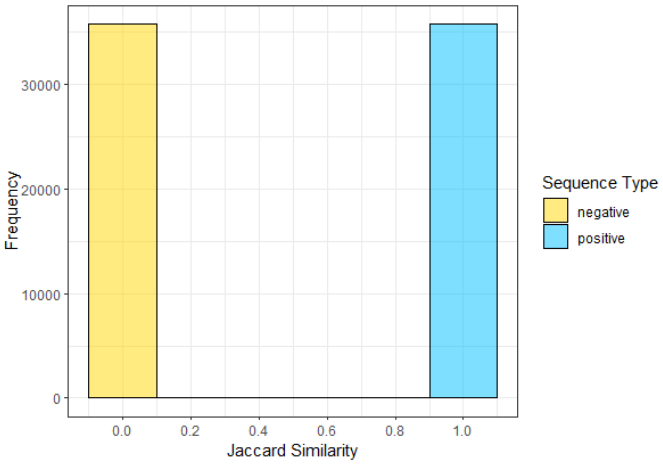

Supplementary Figure 7: Distribution of computed k-mer-based Jaccard similarity scores (k = 3). Yellow = negative, light blue = positive.

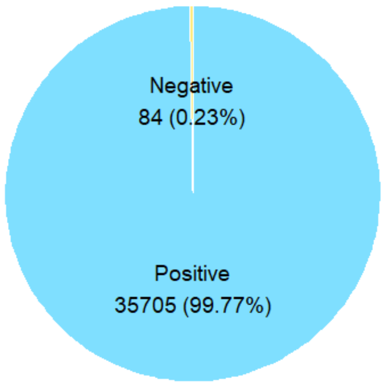

Supplementary Figure 8: Classification of candidate sequences by the Random Forest model. Yellow = negative, light blue = positive.
